# Changes in the Balance Performance of Polish Recreational Skiers after Seven Days of Alpine Skiing

**DOI:** 10.2478/hukin-2014-0108

**Published:** 2014-12-30

**Authors:** Beata Wojtyczek, Małgorzata Pasławska, Christian Raschner

**Affiliations:** 1 Academy of Physical Education, Warsaw, Poland.; 2 College of Tourism and Language Studies, Warsaw, Poland.; 3 Department of Sport Science, University of Innsbruck, Innsbruck, Austria.

**Keywords:** physical activities, winter sport, postural stability, MFT S3-Check

## Abstract

Alpine skiing is one of the most popular leisure time winter sporting activities. Skiing imposes high requirements concerning physical fitness, particularly regarding balance abilities. The main objective of this study was to evaluate the changes in balance performance of recreational skiers after a seven-day ski camp. A total of 78 students - 24 women and 54 men - participated in the study. The ski course was held in accordance with the official program of the Polish Ski Federation. The study sample was comprised of 43 beginners and 35 intermediate skiers. All students were tested with the MFT S3-Check, the day before and the day after the ski camp. The test system consisted of an unstable uniaxial platform, with an integrated sensor and corresponding software. Changes in balance performance (sensory and stability index) were evaluated using paired t-tests. Additionally, changes in sensory and stability categories, which were based on the norm data, were analyzed. Female and male participants showed significantly better sensory and stability indices after skiing. Considerable changes from weak or very weak to average or good balance categories could be seen after skiing for both sexes. Regarding skiing experience, both beginners and intermediate skiers improved their sensory and stability indices significantly after skiing. Hence, recreational alpine skiing resulted in better balance performance regardless of sex or skiing experience. Skiing as an outdoor activity offers the opportunity to improve balance performance with a positive impact on everyday life activities.

## Introduction

The importance of regular exercise for maintaining health and quality of life is a well-known and accepted fact. Physical activity becomes more important in one’s life as more functional capacities decline due to the aging process (Hollmann et al., 2007). For many years, the emphasis in all age groups has been on maintaining or improving endurance and strength. Scientifically evaluated forms of diagnosis and training programs have been developed and used for this purpose. In comparison, much less attention has been paid to coordinative skills such as balance performance, although these skills are fundamental for many everyday life activities. It was shown that sensorimotor deficits could be identified as a fall risk factor, particularly in elderly people (Muir et al., 2010). Moreover, many researchers have reported on reduced balance ability caused by injury or illness (Arnold et al., 2009; Ray et al., 2008).

With millions of participants, alpine skiing is one of the most popular recreational winter sports, regardless of sex, age or a skill level. In 1933, Mülly already wrote about the importance of balance performance in alpine skiing (Mülly, 1933). Since then, the ongoing fascination with alpine skiing has experienced continuous development. This popularity has become more apparent since the introduction of shorter carving skis with more side cut, and these changes have resulted in greater requirements of physical fitness from skiers (Müller and Schwameder, 2003). Modern carving ski technique demands that skiers have a strong sense of sideways and forward/backward balance due to the extensive inward leaning angles of the body and the shorter skis (Raschner et al., 2001). Furthermore, skiers must edge their skis with precision and feeling to carve, requiring optimal sensorimotor abilities. Hrysomallis (2007) reported in his review that, in various sports, poor balance skills were significantly associated with an increased risk of injuries. This association could also be true for alpine skiing. Epidemiological studies have shown a high incidence of serious injuries among adult and adolescent alpine skiers (Burtscher et al., 2008a; Meyers et al., 2007).

To increase individual skiing performance and to reduce the number of skiing injuries in adolescent skiers, greater attention should be paid to the fitness level of this age group (Raschner et al., 2012; Pasławska et al., 2012). It is generally accepted that physical fitness of a high level is responsible for sportive and safe skiing (Turnbull et al., 2009). A number of authors have stated that, in addition to endurance and strength components, coordination and sensorimotor performance are of the utmost importance in alpine skiing (Wölfel et al., 2003; Bambach et al., 2008). Educational facilities, such as schools and academies, require of youths gym lessons or obligatory physical activities, to increase their fitness levels and, hence, their balance abilities. Pasławska et al. (2012) reported that physical fitness testing during school hours could identify those students with low performance, compared to age and sex-matched norms, and could facilitate the initiation of specific intervention programs.

The purpose of the present study was to evaluate the changes in balance performance, relative to sex and skiing experience, of Polish recreational skiers after an academically obligatory seven-day ski camp. We hypothesized that several days of skiing would result in changes in general balance performance due to the specific requirements of this recreational activity.

## Material and Methods

### Participants

The participants included second-year female and male students of the Physical Education Department of the Academy of Physical Education in Warsaw, who participated in an obligatory seven-day winter camp. The ski course was held in accordance with the official program of the Polish Ski Federation (Stanisławski, 2009). A total of 78 students - 24 women and 54 men aged 20–22 years, free of injuries, participated in the research. The age and anthropometric variables of the female and male students are shown in Table 1. The study was conducted the day before and the day after the end of the winter camp, which lasted seven days (six forty-five–minute units of skiing every day). The skiing experience of the participants was as follows: no skiing experience – 43 participants (beginners); and one or more seasons of skiing experience – 35 participants (intermediate skiers). During the three months before the ski camp began, the participants, as a part of obligatory physical activities, participated in one hour of track and field (cross-country running), one hour of martial arts and one hour of team games per week.

The subjects were informed of the risks associated with their participation in the tests and skiing, and they provided informed consent before the study began. Prior approval for the study was provided by the Bioethics Commission of the Academy of Physical Education in Warsaw, and testing was conducted according to the Declaration of Helsinki.

### Test device

The MFT S3-Check (Grosshöflein, Austria) is a test device for functionally assessing body stability and the ability to regulate the sensory motor system in a standing position (Raschner et al., 2009). The test system consists of an unstable uniaxial platform, with an integrated sensor and corresponding software. The software, including the database, was programmed by BITsoft (Bitburg, Germany). The round platform has a diameter of 55 cm and is connected to a base-plate with a horizontal axis. It can be tilted up to 12° to both sides. Movements of the test participant’s center of gravity causes the platform to tilt. This tilt can be captured by a tilt sensor, which has a measuring range of +20° to −20° and accuracy of less than 0.5 %. Data were collected at a sampling rate of 100 Hz and were transmitted to the software via a USB port, which also functioned as a power source (5 V). By simply turning the testing system 90°, two different test directions could be measured. If the axis of rotation corresponded to the sagittal plane, this movement was referred to as left-right measurement.

To assess the current state of an individual’s ability to balance, the test system measured the movements of the platform and calculated the *sensory index*. Movements of the horizontal platform position to the left and right were quantified by the *symmetry index*. Both factors were incorporated into the *stability index,* which provided information on the complex sensory motor skill levels of the test participants, including how well they could balance their bodies. The measurement values of the sensory index and the stability index were graded on a nine-point scale (minimum value 1 = very good, maximum value 9 = very weak). Symmetry was assessed according to three categories: 40:60 to 50:50% movement symmetry indicated no preference for one side of the body; 25:75 to 39:61% indicated a slight preference; and results less than 24:76% indicated a noticeable preference for one movement side.

The S3-Check fulfilled the reliability and validity criteria and is in use in fitness settings and in physiotherapy (Raschner et al., 2009). Additionally, age and sex-related norms were generated from the data of more than 5000 subjects (8–70 years of age) (Raschner et al., 2009).

### Test procedure

Prior to testing, each participant completed a standardized general warm-up, a specific warm-up on a Balance Disc and one familiarization trial on the MFT S3-Check to minimize learning effects. During the test, the task for the participant was to keep the platform as level as possible to the right and left sides. The standard test time was set for 30 s twice, with an interval of 30 s between the two test trials. The software chose the better attempt as the test result. In general, all of the tests were conducted in a quiet environment and with attention paid to the option of individually setting the standing position. The test was conducted without shoes and with a free choice of arm posture.

### Statistical analysis

Statistical analysis was performed using STATISTICA software, version 7.1 (StatSoft, Inc., Tulsa, US). Descriptive analyses of the balance test data are presented as the means and standard deviations (SD). A normality test (Kolmogorov-Smirnov test) was applied, and it revealed normal distribution of our data. Differences in the balance performance data before and after the ski camp were evaluated using paired t-tests. A criterion of p≤0.05 defined statistical significance. Additionally, changes in sensory index and stability index categories, which were based on the norm data (Raschner et al., 2009), were analyzed regarding sex and skiing experience.

## Results

### Sex-specific balance performance changes

**Figure 1:**
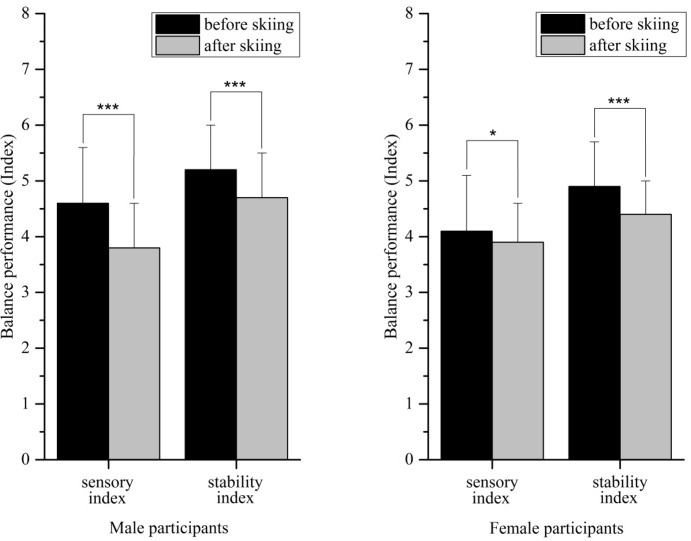
Sensory and stability indices for male and female participants before and after skiing

The sensory and stability indices indicated significant improvements after the seven days of recreational skiing for female and male participants (Figure 1). The symmetry index showed no preference for the right or left side either before or after skiing. Female participants increased their symmetry performance from 4.5% to 0.9% and were more right side–orientated, whereas male participants decreased slightly in symmetry quality from 1.9% before skiing to 4.2% after skiing, with a preference for the right side.

**Figure 2:**
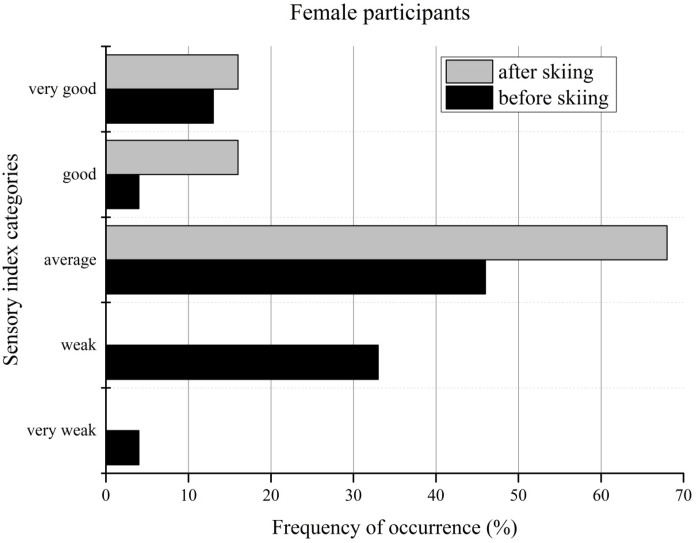
Sensory index categories of female participants before and after the ski camp

**Figure 3:**
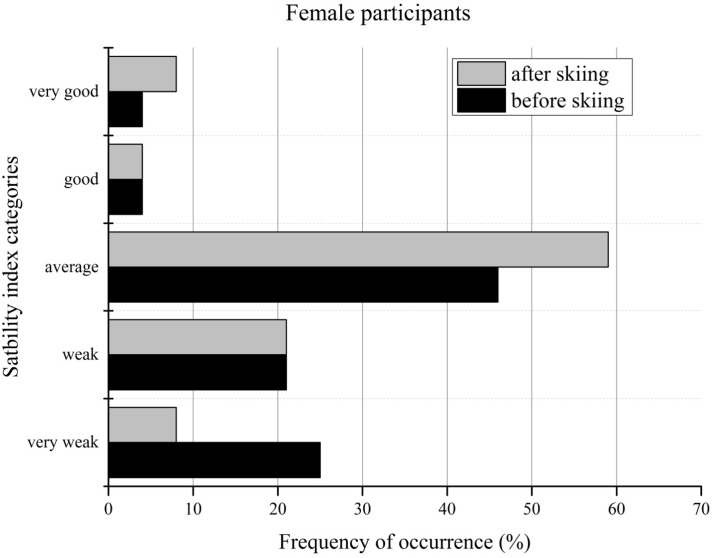
Stability index categories of female participants before and after the ski camp

Figures 2 and 3 compare the sensory and stability indices with the age-related norm data for women, illustrating the changes in the categories - very good to very weak - with regard to the testing date. Before the ski camp, more than one third of the female participants were categorized as weak or very weak by the sensory index. After seven days of skiing, all of the female participants achieved at least average sensory performance, compared to the norm data. The average sensory performance of the female students increased from 46% before skiing to 68% after skiing. The percentage of female recreational skiers who were classified as good or very good increased after the ski camp from 17% to 32%.

Regarding the stability index, almost 46% of the female participants were categorized as weak or very weak before the ski camp and only 8% as good or very good. The greatest changes were noticed in the very weak group, with a decrease of 17%, and in the average group, with an increase of 13% after the ski camp.

**Figure 4:**
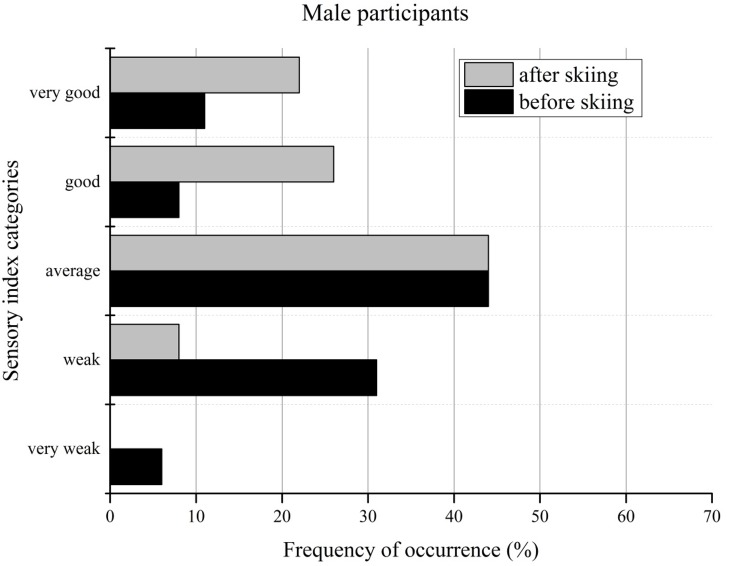
Sensory index categories of male participants before and after the ski camp

**Figure 5:**
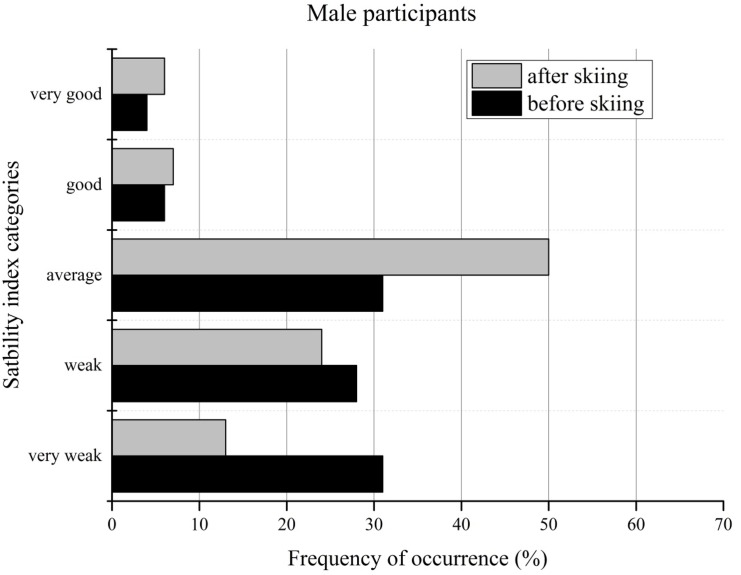
Stability index categories of male participants before and after the ski camp

The effects of skiing on changes in the balance performance categories of male recreational skiers are shown in Figures 4 and 5. Very weak and weak performance on the sensory index decreased clearly from 37% to 8%, whereas the number of participants in the good or very good categories increased from 19% to 48%. Due to this performance change to better results, the comparison of the average category with the reference data remained unchanged at 44%.

The changes in the stability index categories of male participants before and after the ski camp were in agreement with the results of female participants. Compared to the given norm data, the male Polish participants achieved approximately 30% each of very weak, weak or average results before the ski camp. Only 4% and 6% performed good or very good, respectively. Figure 5 shows an increase in right/left stability performance after the ski camp in all of the performance categories. The number of male participants performing at an average level increased from 31% before the ski camp to 50% after skiing.

### Ski experience–specific balance performance changes

Changes in the sensory and stability indices of the beginners and intermediate skiers before and after the ski camp are shown in Table 2. Both groups significantly improved their balance performance after seven days of skiing on the MFT S3-Check. The symmetry index indicated no relevant changes before versus after skiing for either group.

Table 3 shows changes in the sensory and stability index categories of the beginners with no skiing experience and the intermediate skiers before and after the ski camp. The number of beginners with weak or very weak results decreased obviously in the sensory index categories, compared to the norm data. Almost 30% of the subjects were in the good category after the ski camp, compared to 7% before the seven days of skiing; however, slightly fewer participants accomplished the very good category. Twelve percent more of the beginners reached the category of average after the seven days of skiing, compared with the results before skiing. Regarding the stability index, beginners were able to increase the proportion of average results from 28% to 56% after the ski camp, at the expense of weak and very weak results. The good and very good results remained constant.

The results of the sensory indices of the experienced skiers showed that after the end of the ski training, none of the participants had very weak or weak results, compared to the reference data. Experienced skiers increased in the average category to 54%, the good results increased to 17%, and very good sensory performance increased to 29%. Fifty-seven percent of all of the experienced skiers achieved average results in the stability index after skiing, compared to 48% before skiing. A clear decrease in very weak performance resulted in a minor increase in the number of participants in the weak category. The good and very good categories slightly increased after skiing.

## Discussion

It has been reported in the literature that participation in physical activity during childhood and adolescence is a key factor in promoting a more active lifestyle in adulthood, with its associated health benefits (Aarino et al., 2002). Therefore, offering of a broad spectrum of physical activities in schools and academic educational institutions has been strongly recommended. One of these activities could be alpine skiing, which is one of most popular winter sports worldwide in all age groups. It is generally accepted that alpine skiing is a complex sport with a great demand for physical conditioning, involving the cardiorespiratory, neuromuscular and sensorimotor systems (Kahn et al., 1996; Müller and Schwameder, 2003; Malliou et al., 2004; Krautgasser et al., 2009). Alpine skiing is undertaken in a highly challenging and changing environment, requiring optimal postural control. Taube and Gollhofer (2012) emphasized that alpine skiers must rapidly compensate for internal and external forces under highly dynamic conditions to avoid loss of balance. A skier must adjust sideways and maintain fore-aft balance in turning and gliding. He or she is in balance and will not fall as long as the total of all of the forces acting on the center of gravity passes through the body’s base of support (LeMaster, 2010). It is necessary during skiing to make small adjustments constantly in response, e.g., to changes in speed, turning radius, terrain or snow conditions.

Novices ski with their feet apart to have greater leeway for balance regulation, whereas experts ski in a narrower ski position due to their better ski-specific balance, or they can vary the width of their stance relative to the slope conditions and their speed. Experts know that an unstable upper body during skiing can also have a negative effect on a skier’s balance. Therefore, regardless of skiing experience, the so-called neutral upper body position of a skier has been emphasized.

The better the performance is, the greater the demand is that skiers have a strong sense of balance and edging precision of the skis. These requirements have been confirmed by biomechanical studies, in which carving turns showed more radical inward leaning and more ski edging (Raschner et al., 2001). The shorter and more specifically articulated carving skis demand better sagittal balance ability with an optimal proprioceptive mechanism to remain centrally positioned over the skis.

The present study showed that seven days of recreational alpine skiing, as part of an obligatory winter camp, improved balance performance in Polish students regardless of sex or skiing experience. Female and male participants showed significantly lower sensory and stability indices after skiing, indicating better performance of sideways balance after skiing. In particular, those participants whose performance was weak or very weak before skiing improved their postural control after skiing. The symmetry index indicated no preference of the participants for the right or left side before skiing, and this observation did not change after skiing. This finding was not surprising because the participants were students of the Academy of Physical Education with the expected right/left balanced motor control. Balance performance was evaluated with the MFT S3 Check, which has been regularly used in performance testing of Austrian adolescent ski racers to monitor and predict their postural control (Raschner et al., 2013). It is a dynamic test that challenges ski-specific sensorimotor requirements better than a static test.

Our results were in agreement with the scarce information available regarding balance changes during or after a ski training camp. Hydren et al. (2013) investigated the effects of a weeklong high-altitude ski training camp (living at 2800 m, skiing up to 3800 m) on fitness performance of young lowland ski racers. The authors showed that balance performance, evaluated with the Y-Balance test, improved after three days of skiing. It was noted that physical performance changes which might be variable, depended on training status, body mass changes and altitude sickness symptoms. In particular, hydration should be encouraged throughout the time at high-altitude ski camps.

The authors of the present study are aware of only one study that addressed the impact of additional balance training on skiing performance over two weeks at a skiing camp (Malliou et al., 2004). Skiing, coupled with additional indoor balance training in the afternoon, improved downhill agility ski test results significantly better than skiing only, in which a control group participated. Thus, specific indoor balance training was helpful in improving ski technique over two weeks at this skiing camp.

Skiers are normally exposed to low temperatures over several hours, which can affect the cardiovascular and nervous systems, as well as physical and cognitive performance (Burtscher et al., 2008b). It was shown that reduced sensitivity of the mechanoreceptors in the skin of the feet, caused hypothermic anesthesia, impaired to some extent the ability to maintain postural control (Stal et al., 2003). This effect should be considered because decreased postural control during skiing, coupled with muscle fatigue, could increase injury risk.

Additionally, low vision during skiing, caused by fog or snowfall, can affect the balance performance of leisure-time skiers. For example, unexpected changes in slope conditions, such as icy areas or bumps, often lead to balance problems and falls.

The balance tests in this study were performed without any shoes. It must be mentioned that the stiff ski boots of skiers facilitate the transfer of power to the skis, but they also increase the difficulty in maintaining postural control. Mildner et al. (2010) showed that balance performance on the MFT S3-Check was negatively influenced when wearing ski boots. In particular, beginners should avoid ski boots that are overly stiff because they can limit ankle joint movements, which are important for balance regulation during skiing. In this context, a study by Noé et al. (2009) found that mechanical effects of wearing ski boots resulted in changes in postural strategy through the reorganization of muscle coordination in experienced skiers.

The improvements in balance performance in our study could also be explained by guided skiing including a number of lateral and fore-aft drills over a week of skiing. Exercises such as skiing only on the outside ski with the inside leg raised or skiing without poles are part of the curricula of ski instructor associations.

Dry land coordination training, which challenges a person’s balance on wobble boards or other similar equipment prior to skiing, has also been strongly recommended. These recommendations have been confirmed by several studies that have demonstrated beneficial effects on postural control and injury prevention (e.g., Hübscher et al., 2010). In this context, Taube and Gollhofer (2012) speculated that balance-trained subjects could optimize the coordination of their movements so that they might be able to avoid critical joint positions, which are crucial for recreational skiers.

There were some limitations of our study, and the data must be interpreted in this context. First, we were unable to measure fore-aft balance performance, which is also very important for skiers. Nevertheless, internal calculations showed a very strong correlation of sideways and fore-aft balance in young Austrian ski racers. It could be speculated that this association is also true for recreational skiers. Second, we were not able to include a control group of recreational skiers in whom we could measure balance performance changes without skiing over this time period. However, it can be assumed that changes in balance performance of a control group would have been minor over an eight-day time period.

In conclusion, regardless of sex or ski experience, we found substantial improvements in balance performance of physically active young people after seven days of supervised alpine skiing with experts. In a recently published study, Ruedl et al. (2014) stated that the most frequent causes of injuries of skiers and snowboarders were self-inflicted falls (87 %). The chance of self-inflicted falls is reduced as a skier’s balance improves. We suggest that beginning skiers join ski school lessons for supervised skiing. Skiing with experts provides a structured framework of different balance exercises on snow. In general skiing offers a great opportunity in nature to increase the skier’s fitness level, in particular his or her coordination.

## Figures and Tables

**Table 1:** Age and anthropometric parameters of the female and male participants

**Sex**	**Age (years)**	**Body height (cm)**	**Body mass (kg)**	**BMI (kg/m^2^)**
F (n=24)	20.5 ± 0.76	169 ± 7.73	63 ± 7.80	22 ± 1.86
M (n=54)	20.5 ± 0.81	182 ± 6.51	80 ± 9.62	24 ± 2.26

F = female participants, M = male participants; mean ± standard deviation

**Table 2:** Stability and sensory indices of the beginners and intermediate skiers before and after the ski camp

**Ski experience**	**Balance performance**	**Before skiing**	**After skiing**	**p**
Beginners (n=43)	Sensory index	4.34 ± 1.09	3.92 ± 0.80	p<0.05
	Stability index	5.04 ± 0.95	4.65 ± 0.74	p<0.001

Intermediate skiers (n=35)	Sensory index	4.56 ± 0.87	3.63 ± 0.81	p<0.001
	Stability index	5.09 ± 0.67	4.55 ± 0.80	p<0.001

Stability and sensory indices as the means ± standard deviations

**Table 3:** Sensory and stability index categories of beginners and intermediate skiers before and after the ski camp

**Ski experience**	**Index categories**	**Before/after skiing**	**Very good**	**Good**	**Average**	**Weak**	**Very weak**
**Beginners**	Sensory index	Before	21	7	37	26	9
		After	14	28	49	9	0

	Stability index	Before	5	9	28	30	28
		After	5	9	56	21	9

**Intermediate skiers**	Sensory index	Before	9	9	48	31	3
		After	29	17	54	0	0

	Stability index	Before	3	3	48	20	26
		After	6	6	57	22	9

Changes in sensory and stability index categories: very good, good, average, weak and very weak in percentages
